# Protective Effects of Sodium Copper Chlorophyllin and/or Ascorbic Acid Against Barium Chloride-Induced Oxidative Stress in Mouse Brain and Liver

**DOI:** 10.3390/molecules30153231

**Published:** 2025-08-01

**Authors:** Salma Benayad, Basma Es-Sai, Yassir Laaziouez, Soufiane Rabbaa, Hicham Wahnou, Habiba Bouchab, Hicham El Attar, Bouchra Benabdelkhalek, Loubna Amahdar, Oualid Abboussi, Raphaël Emmanuel Duval, Riad El Kebbaj, Youness Limami

**Affiliations:** 1 Sciences and Engineering of Biomedicals, Biophysics and Health Laboratory, Higher Institute of Health Sciences, Hassan First University, Settat 26000, Morocco; salmabenayad6@gmail.com (S.B.); essaibassma1999pfe@gmail.com (B.E.-S.); y.laaziouez@gmail.com (Y.L.); rabbaasoufian@gmail.com (S.R.); hwwahnou@gmail.com (H.W.); habibabouchab78@gmail.com (H.B.); loubna.amahdar@uhp.ac.ma (L.A.); youness.limami@gmail.com (Y.L.); 2 Laboratory of Integrative Biology, Faculty of Sciences Ain Chock, Hassan II University, B.P 2693, Maarif, Casablanca 20100, Morocco; 3 Higher Institute of Nursing Professions and Technical Health (ISPITS), Errachidia 52000, Morocco; 4 Annasr Pathology Center, El Jadida 24000, Morocco; cpa86513@gmail.com; 5 Centre de Médecine Régénérative et Intégrative RegenCab, Tétouan 93000, Morocco; dr.b.benabdelkhalek.mestari@gmail.com; 6 Physiology and Physiopathology Team, Faculty of Sciences, Genomic of Human Pathologies Research Centre, Mohammed V University, Rabat 1014, Morocco; o.abboussi@um5r.ac.ma; 7 Université de Lorraine, F-54000 Nancy, France

**Keywords:** barium chloride, oxidative stress, sodium copper chlorophyllin, ascorbic acid, liver, brain, lipid peroxidation, antioxidant enzymes

## Abstract

Barium chloride (BaCl_2_), a known environmental pollutant, induces organ-specific oxidative stress through disruption of redox homeostasis. This study evaluated the protective effects and safety profile of sodium copper chlorophyllin (SCC) and ascorbic acid (ASC) against BaCl_2_-induced oxidative damage in the liver and brain of mice using a two-phase experimental protocol. Animals received either SCC (40 mg/kg), ASC (160 mg/kg), or their combination for 14 days prior to BaCl_2_ exposure (150 mg/L in drinking water for 7 days), allowing evaluation of both preventive and therapeutic effects. Toxicological and behavioral assessments confirmed the absence of systemic toxicity or neurobehavioral alterations following supplementation. Body weight, liver and kidney indices, and biochemical markers (Aspartate Aminotransferase (ASAT), Alanine Aminotransferase (ALAT), creatinine) remained within physiological ranges, and no anxiogenic or locomotor effects were observed. In the brain, BaCl_2_ exposure significantly increased SOD (+49%), CAT (+66%), GPx (+24%), and GSH (+26%) compared to controls, reflecting a robust compensatory antioxidant response. Although lipid peroxidation (MDA) showed a non-significant increase, SCC, ASC, and their combination reduced MDA levels by 42%, 37%, and 55%, respectively. These treatments normalized antioxidant enzyme activities and GSH, indicating an effective neuroprotective effect. In contrast, the liver exhibited a different oxidative profile. BaCl_2_ exposure increased MDA levels by 80% and GSH by 34%, with no activation of SOD, CAT, or GPx. Histological analysis revealed extensive hepatocellular necrosis, vacuolization, and inflammatory infiltration. SCC significantly reduced hepatic MDA by 39% and preserved tissue architecture, while ASC alone or combined with SCC exacerbated inflammation and depleted hepatic GSH by 71% and 78%, respectively, relative to BaCl_2_-exposed controls. Collectively, these results highlight a differential, organ-specific response to BaCl_2_-induced oxidative stress and the therapeutic potential of SCC and ASC. SCC emerged as a safer and more effective agent, particularly in hepatic protection, while both antioxidants demonstrated neuroprotective effects when used individually or in combination.

## 1. Introduction

Among the many environmental pollutants to which humans are exposed on a daily basis, heavy metals occupy a key position due to their persistent toxicity and bioaccumulation in living organisms. One such agent is barium, an alkaline earth metal. Although it occurs naturally in the environment, it can become a serious health threat when released in the form of soluble salts, particularly in industrial processes such as mining, drilling, and the manufacture of paper and textiles [1]. When in soluble form, barium compounds, particularly barium chloride (BaCl_2_), can contaminate drinking water and food, which are the main human exposure sources [2]. Although less studied than other metals, acute or chronic exposure to BaCl_2_ can lead to significant cellular and tissular injury. When absorbed, BaCl_2_ splits into barium ions (Ba^2+^), which are highly toxic, and chloride ions (Cl^−^), which are generally less harmful and are excreted by the kidneys [3]. Ba^2+^ may then distribute to many tissues, including the liver, muscles, kidneys, heart, and nervous system, triggering a wide range of clinical responses, from gastrointestinal disorders to severe cardiovascular and neuromuscular damage [4]. The systemic toxicity observed is often associated with ionic imbalance, but also with more complex cellular processes such as oxidative stress [2]. BaCl_2_-induced oxidative stress is mainly attributed to its ability to destabilize the balance between reactive oxygen species (ROS) generation and the intrinsic antioxidant defense systems [2]. Although reactive nitrogen species (RNS) may also contribute to cellular damage under oxidative conditions, their specific involvement in BaCl_2_ toxicity remains unclear and requires further investigation. From a structural and functional point of view, Ba^2+^ may disrupt the mitochondrial electron transport chain (ETC) [5], promoting electron leakage and excessive formation of superoxide anions (O_2_•^−^). Such reactive intermediates trigger a chain reaction of oxidative damage that affects vital cellular components, including membrane lipids, structural and functional proteins, and nucleic acids, leading to protein and lipid peroxidation and the production of cytotoxic aldehydes such as malondialdehyde (MDA) and 4-hydroxynonenal (4-HNE), further exacerbating cellular damage. Meanwhile, Ba^2+^ compromises antioxidant defenses by inhibiting key enzymes, such as superoxide dismutase (SOD), catalase (CAT), or glutathione peroxidase (GPx), thereby reducing ROS elimination and amplifying oxidative damage. These genomic, metabolic, and functional alterations caused by oxidative stress have been implicated in the onset or progression of several diseases [6]. This concept has also been highlighted in recent multi-omics studies exploring oxidative and inflammatory pathways in unrelated pathological conditions [7].

Because oxidative stress has a pervasive impact at the cellular level, organs with high metabolic demands and complex physiological roles are particularly vulnerable. The liver and brain, among others, are notable for their increased exposure to oxidative stress. The liver, as the main detoxification organ, is constantly exposed to xenobiotics [8], while the brain, characterized by relatively lower levels of enzymatic antioxidants and high oxygen consumption, is especially sensitive to this oxidative imbalance [9]. Therefore, maintaining redox balance is vital for preserving their function and structural integrity.

**Figure 1:**
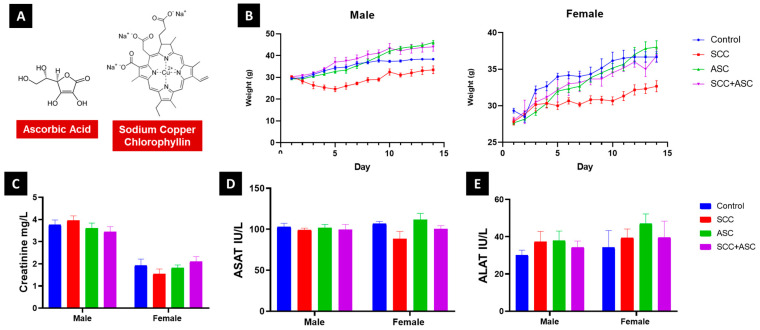
(**A**). Ascorbic acid and sodium copper chlorophyllin molecules. (**B**). Body weight variations of male and female and plasma levels. (**C**). Creatinine (**D**). ASAT and (**E**) ALAT of male and female mice following subacute treatment with SCC (40 mg/kg), ASC (160 mg/kg), or their combination. Data are expressed as mean ± SD.

To counteract these deleterious effects, there is growing interest in nature-derived molecules with antioxidant properties due to their ability to neutralize ROS and enhance endogenous antioxidant defenses [10,11,12,13,14,15,16]. In this light, much interest has been given to sodium copper chlorophyllin (SCC), a semi-synthetic, water-soluble derivative of chlorophyll with potent metal-chelating properties, and ascorbic acid (ASC, i.e., vitamin C), a well-established natural antioxidant (Figure 1A). SCC exhibits unique dual functionality, acting both as a direct free radical scavenger and as a transition metal chelator, particularly effective against copper- and iron-mediated Fenton reactions. Its stable porphyrin structure allows for efficient electron delocalization, making it particularly effective against a broad spectrum of reactive oxygen species [17].

ASC, abundantly present in fruits and vegetables, serves as the primary aqueous-phase antioxidant in biological systems. It functions through multiple mechanisms [18]: (1) direct scavenging of reactive oxygen and nitrogen species; (2) regeneration of other antioxidants like vitamin E from their oxidized forms; and (3) modulation of enzymatic antioxidant systems through redox signaling. However, it is important to note that ASC can exhibit prooxidant activity under certain conditions, particularly in the presence of free transition metals where it may participate in redox cycling and generate additional oxidative species [18]. Furthermore, ASC and SCC has been widely studied for its protective effects against oxidative stress induced by heavy metals, drugs, and environmental toxins [19,20]. SCC is a semi-synthetic, water-soluble derivative of chlorophyll, the green pigment found in plants and algae [21]. It exhibits a potent antioxidant effect by neutralizing ROS and inhibiting lipid peroxidation [22]. Furthermore, it has metal-chelating properties, particularly toward transition metals such as copper and iron, which catalyze ROS production through the Fenton reaction [23]. Whether SCC interacts directly with barium ions remains to be clarified.

This study hypothesized that SCC and ASC would mitigate BaCl_2_-induced oxidative stress in an organ-specific manner, with SCC expected to offer stronger protection due to its metal-chelating properties. This research aimed to examine BaCl_2_’s oxidative effects in the liver and brain while assessing the protective roles of SCC and ASC. Using a combined biochemical and histopathological approach, this study analyzed oxidative stress markers and tissue structure to uncover the mechanisms behind these antioxidants’ effects. The findings could guide future interventions against BaCl_2_ toxicity.

## 2. Results

### 2.1. Safety Profile and Behavioral Effects of SCC and ASC

#### 2.1.1. SCC and ASC Toxicity

No significant differences in body weight were observed across treatment groups during the 14-day regimen (Figure 1B). Macroscopic inspection of liver and kidneys revealed no visible abnormalities. Organ weight indices were not significantly altered for either liver or kidneys following treatment. Biochemical analysis showed no significant elevation or suppression of plasma creatinine, ASAT, ALAT levels in treated groups compared to controls (Figure 1C–E). All values remained within physiological ranges, indicating no marked hepatotoxicity or nephrotoxicity.

#### 2.1.2. SCC and ASC Effect on Behavior

**Figure 2:**
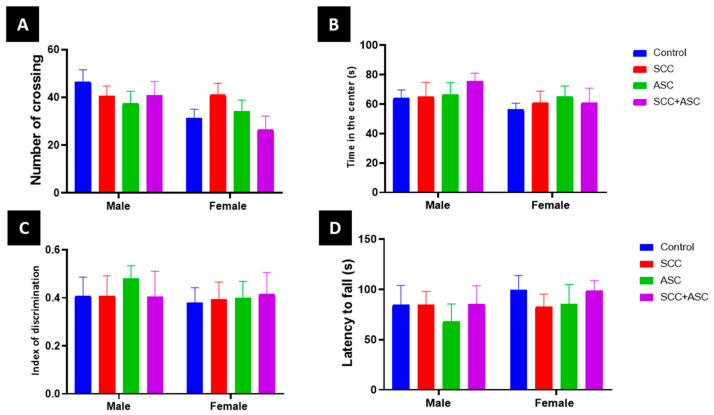
Effect of subacute SCC (40 mg/kg), ASC (160 mg/kg), or their combination on male and female mice behavior including (**A**) locomotor activity; (**B**) anxiety-like behavior, (**C**) episodic memory as measured by discrimination index in the novel object recognition test, and (**D**) motor performance (rotarod test)**.** Data are expressed as mean ± SD.

Open field analysis demonstrated that subacute administration of SCC, ASC, or their combination did not induce significant anxiogenic or locomotor effects in mice (Figure 2A). Two-way ANOVA revealed no significant impact of treatment on time spent in the center (*F* = 0.28, *p* = 0.891) or on the number of line crossings (*F* = 0.64, *p* = 0.635), and no significant treatment-by-sex interaction was observed for either parameter (center time: *F* = 0.29, *p* = 0.885; crossings: *F* = 1.07, *p* = 0.382) (Figure 2B). However, a significant main effect of sex was noted for locomotor activity (crossings: *F* = 8.46, *p* = 0.0054), indicating higher activity in one sex, while center time remained unaffected (*F* = 1.05, *p* = 0.31). Cognitive performance, assessed by the novel object recognition test, showed no significant differences in the discrimination index based on treatment (*F* = 0.12, *p* = 0.976), sex (*F* = 1.25, *p* = 0.270), or their interaction (*F* = 0.36, *p* =0.833) (Figure 2C). Similarly, motor coordination evaluated using the rotarod test remained unaltered across groups, with no significant effects of treatment (*F* = 0.38, *p* = 0.821), sex (*F* = 1.08, *p* = 0.303), or their interaction (*F* = 0.13, *p* = 0.972) on latency to fall (Figure 2D).

### 2.2. Effects on Body Weight and Relative Weight

**Figure 3:**
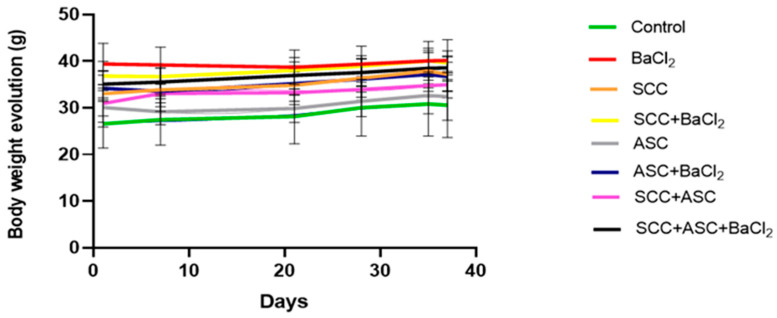
Evolution of body weight (g) in mice for each experimental groups during the 36-day treatment period. *n* = 4 for BaCl_2_-untreated (*(−) BaCl*_2_) groups and *n* = 6 for BaCl_2_-treated (*(+) BaCl*_2_) BaCl_2_ groups. Data are expressed as mean ± SD.

During the entire experimental period, the body weight of the mice was monitored regularly to assess general health and potential systemic effects of the treatments. As shown in Figure 3, all groups showed a relatively stable trend in body weight over time, with no marked fluctuations. The groups receiving SCC, ASC or their combinations, alone or in co-administration with BaCl_2_, showed no significant weight loss (*p* > 0.05).

**Figure 4:**
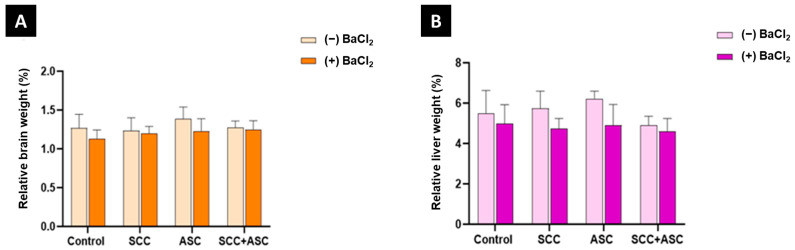
(**A**) Relative brain weight (% of body weight); (**B**) relative liver weight (% of body weight) for each experimental group: control, SCC, ASC, and SCC + ASC, under both BaCl_2_-untreated (*(−) BaCl*_2_) and BaCl_2_-treated (*(+) BaCl*_2_) conditions. *n* = 4 for (−) BaCl_2_ groups and *n* = 6 for (+) BaCl_2_ groups. Data are expressed as mean ± SD.

As shown in Figure 4A, exposure to BaCl_2_ had no significant effect on relative brain weight. Similarly, administration of SCC, ASC, or their co-administration did not result in any significant changes in brain mass (*p* > 0.05). At the same time, no significant changes were observed in relative liver weight in any group, regardless of BaCl_2_ exposure or treatments (*p* > 0.05), as shown in Figure 4B.

### 2.3. Serum Biochemical Markers

Among all the serum biochemical and ionic parameters assessed, statistically significant changes were observed only in the ASC + BaCl_2_ group. In this group, triglyceride levels were significantly increased compared to the BaCl_2_ group (*p* < 0.05), and ASAT activity was significantly higher compared to both the negative control (*p* < 0.05) and the BaCl_2_ group (*p* < 0.05). No statistically significant differences were detected for any other parameter in the remaining experimental groups (Table 1).

### 2.4. Effect on Cerebral Antioxidant Markers

**Figure 5:**
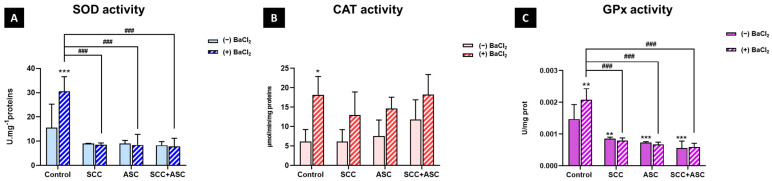
(**A**): Superoxide dismutase (SOD) activity; (**B**) catalase (CAT) activity; and (**C**): glutathione peroxidase (GPx) activities were measured in brain homogenates of mice from each experimental group: control, SCC, ASC, and SCC + ASC, under both BaCl_2_-untreated (*(−) BaCl*_2_) and BaCl_2_-treated (*(+) BaCl*_2_) conditions. Data are expressed as mean ± SD. *n* = 4 for (−) BaCl_2_ groups and *n* = 6 for (+) BaCl_2_ groups. Statistical significance: ^###^ p < 0.001 vs. (+) BaCl_2_ control group, * p < 0.05, ** p < 0.01, *** p < 0.001 vs. (−) BaCl_2_ control group.

To evaluate the antioxidant effects of SCC and/or ASC, we measured key biomarkers of oxidative stress. SOD activity, the first enzymatic line of defense against oxidative stress, was significantly increased following BaCl_2_ exposure compared to the negative control group (*p* < 0.001). However, treatment with SCC, ASC, or both reversed this increase (*p* < 0.001), bringing SOD levels back to values comparable to the basal control group. Furthermore, no significant change was observed in the treatments alone compared to the control group (Figure 5A). CAT activity showed an increase with BaCl_2_ treatment. However, although not significant, an increased CAT activity was observed among ASC and CHL + ASC-treated groups in BaCl_2_-untreated mice (Figure 5B). Additionally, the results showed a significant increase (*p* < 0.01) in GPx activity in the BaCl_2_-treated control group compared to the BaCl_2_-untreated control group. At the same time, a significant decrease was observed in the SCC + BaCl_2_ group (*p* < 0.001), the ASC + BaCl_2_ group (*p* < 0.001), and the dual treatment + BaCl_2_ group (*p* < 0.001) compared to the BaCl_2_-treated control group. Treatments alone also decreased GPx activity compared to the negative control (*p* < 0.01 for the SCC alone group and *p* < 0.001 for the ASC alone group and the combination alone group), as shown in Figure 5C.

**Figure 6:**
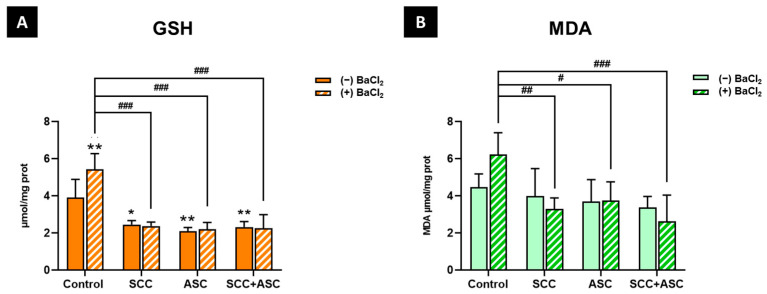
(**A**) GSH and (**B**) MDA levels were measured in brain homogenates of mice from each experimental group: control, SCC, ASC, and SCC + ASC, under both BaCl_2_-untreated (*(−) BaCl*_2_) and BaCl_2_-treated (*(+) BaCl*_2_) conditions. Data are expressed as mean ± SD. *n* = 4 for (−) BaCl_2_ groups and *n* = 6 for (+) BaCl_2_ groups. Statistical significance: * p < 0.05, ** p < 0.01 vs. (−) BaCl_2_ control group, ^#^ p < 0.05, ^##^ p < 0.01, ^###^ p < 0.001 vs. (+) BaCl_2_ control group.

As for non-enzymatic markers, the GSH levels were significantly increased (*p* < 0.01) following BaCl_2_ exposure compared to the negative control group. Also, a significant decrease in GSH levels was observed in the SCC + BaCl_2_ group (*p* < 0.001), the ASC+ BaCl_2_ group (*p* < 0.001), and the combination + BaCl_2_ group (*p* < 0.001) compared to the positive control group. Moreover, treatments alone (SCC, ASC and SCC + ASC) showed a significant decrease in GSH levels compared to the negative control group (*p* < 0.05, *p* < 0.01, *p* < 0.01, respectively) (Figure 6A). MDA, a well-established marker of lipid peroxidation and oxidative damage on cell membranes, was slightly increased in response to BaCl_2_ exposure, although this change was not statistically significant. However, the administration with SCC (*p* < 0.01), ASC (*p* < 0.05), or their co-treatment (*p* < 0.001) alongside BaCl_2_ significantly reduced MDA levels compared to the BaCl_2_-only group. Additionally, all the groups in the absence of BaCl_2_ did not significantly affect MDA levels compared to the control group (Figure 6B).

### 2.5. Effects on Hepatic Antioxidants Markers

**Figure 7:**
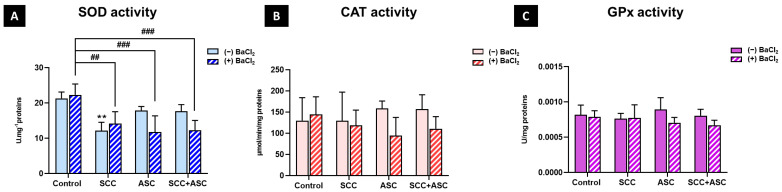
(**A**) Superoxide dismutase (SOD) activity; (**B**) catalase (CAT) activity; and (**C**) glutathione peroxidase (GPx) were measured in liver homogenates of mice from each experimental group: control, SCC, ASC, and SCC + ASC, under both BaCl_2_-untreated (*(−) BaCl*_2_) and BaCl_2_-treated (*(+) BaCl*_2_) conditions. Data are expressed as mean ± SD. *n* = 4 for (−) BaCl_2_ groups and *n* = 6 for (+) BaCl_2_ groups. Statistical significance: ^##^ p < 0.01, ^###^ p < 0.001 vs. (+) BaCl_2_ control group, ** p < 0.01.

As illustrated in Figure 7B,C, no statistically significant differences between experimental groups were observed, neither for catalase activity nor GPx activity, in liver. However, hepatic SOD activity was significantly reduced in the SCC group compared with the control group (*p* < 0.01). Moreover, a significant decrease in SOD activity was observed in the SCC + BaCl_2_ (*p* < 0.01), ASC + BaCl_2_ (*p* < 0.001) as well as the SCC + ASC + BaCl_2_ (*p* < 0.001) groups, compared to the BaCl_2_ group Figure 7A.

**Figure 8:**
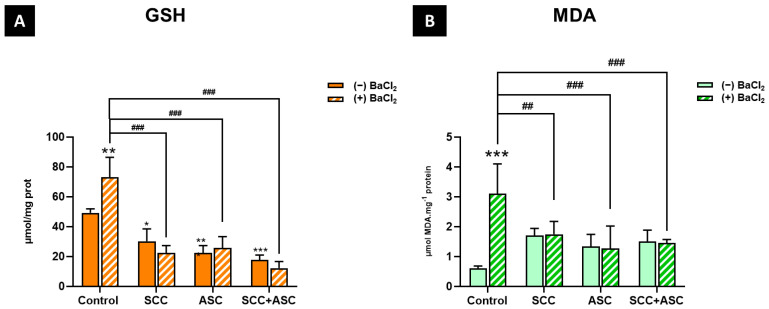
(**A**): GSH and (**B**) MDA levels were measured in liver homogenates of mice from each experimental group: control, SCC, ASC, and SCC + ASC, under both BaCl_2_-untreated (*(−) BaCl*_2_) and BaCl_2_-treated (*(+) BaCl*_2_) conditions. Data are expressed as mean ± SD. n = 4 for Ba^−^ groups and n = 6 for Ba^+^ groups. Statistical significance: * p < 0.05, ** p < 0.01, *** p < 0.001 vs. (−) BaCl_2_ control group, ^##^ p < 0.01, ^###^ p < 0.001 vs. (+) BaCl_2_ control group.

GSH levels were significantly increased following BaCl_2_ exposure (*p* < 0.01) compared to the negative control group. Furthermore, a significant decrease in GSH levels was observed in the SCC + BaCl_2_ group (*p* < 0.001), the ASC + BaCl_2_ group (*p* < 0.001), and the combination + BaCl_2_ group (*p* < 0.001) compared to the positive control group. The treatments alone (SCC, ASC, and SCC + ASC) showed a significant decrease in GSH levels compared to the negative control group (*p* < 0.05, *p* < 0.001, *p* < 0.001, respectively) Figure 8A. As for the MDA levels, a significant increase in the BaCl_2_-exposed group compared to the control group (*p* < 0.001) was observed. However, BaCl_2_ groups treated with SCC, ASC, or both treatments significantly reduced MDA levels compared to the BaCl_2_-only group (*p* < 0.01 and *p* < 0.001, respectively). No significant difference in MDA levels was observed between the treatments alone and the negative control group (Figure 8B).

### 2.6. Histopathological Examination

**Figure 9:**
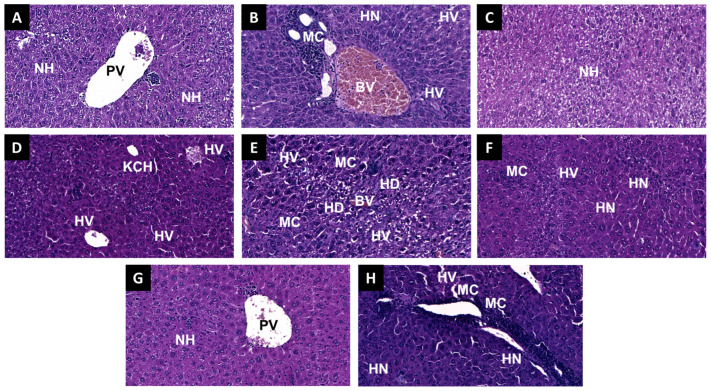
Representative histological images of liver tissue from the different experimental groups (H&E staining, original magnification ×20). (**A**) Control; (**B**) BaCl_2_; (**C**) SCC; (**D**) BaCl_2_ + SCC; (**E**) ASC; (**F**) BaCl_2_ + ASC; (**G**) SCC + ASC; (**H**) BaCl_2_ + SCC + ASC. HN: hepatocellular necrosis, (characterized by loss of cellular architecture, cytoplasmic homogenization, and absence of nuclei); HV: hepatocellular vacuolization, (indicating cytoplasmic rarefaction and mild cellular edema); MC: mononuclear cell (infiltrating inflammatory site); KCH: Kupffer cell hyperplasia (within hepatic sinusoids); HD: hepatocellular distress, (with swollen hepatocytes exhibiting cytoplasmic pallor and preserved nuclei); BV: blood vessel; PV: portal vein.

Microscopic examination of the negative control group revealed a normal liver architecture with well-preserved hepatocytes and intact sinusoidal structures (Figure 9A). Similarly, the SCC-treated group and the SCC + ASC group displayed a normal histological appearance (Figure 9C) (Figure 9G), while the ASC-treated group exhibited mild centrilobular hepatocellular distress with moderate vacuolization (Figure 9E). Following BaCl_2_ exposure, significant hepatic disruption was observed, including a large central necrotic area, cytoplasmic disintegration, peri-necrotic vacuolization, moderate swelling, and prominent mononuclear infiltration (Figure 9B). Also, the BaCl_2_ + ASC group exhibited more severe hepatic damage, including extensive vacuolization, multiple necrotic foci, and dense inflammatory infiltration (Figure 9F). Additionally, the BaCl_2_ + SCC + ASC combination resulted in pronounced pathological alterations, such as extensive perivascular and periportal inflammation, focal necrosis, hepatocyte vacuolization, and sinusoidal congestion (Figure 9H). In contrast, in the BaCl_2_ + SCC group, hepatocyte plates remained largely intact, with mild vacuolization and moderate Kupffer cell hyperplasia but without confluent necrosis (Figure 9D).

## 3. Discussion

Understanding the mechanisms underlying BaCl_2_-induced toxicity and the potential efficiency of antioxidant interventions remains a major focus in the field of environmental toxicology. In this context, the present study investigated the impact of BaCl_2_ exposure on cerebral and hepatic oxidative stress and histopathological alterations, as well as the potential protective effects of SCC and ASC, or both.

In contrast to some reports indicating that subacute BaCl_2_ exposure may lead to marked weight loss and clinical signs of toxicity [24], our study did not reveal any significant decreases in body weight among the groups, suggesting that under the present experimental conditions, neither BaCl_2_ exposure nor antioxidant treatments affected the general condition or feeding behavior of the animals. Similarly, no significant changes were observed in the relative brain weight across the experimental groups. This result indicates that the treatments applied did not produce overt neurotoxic effects sufficient to alter brain mass under the present experimental conditions. Moreover, the relative liver weight also remained unchanged in all groups, throughout the duration of our experiments. However, among all serum parameters evaluated, significant alterations were observed only in the ASC + BaCl_2_ group, which showed elevated triglyceride levels compared to the BaCl_2_ group and a significant increase in ASAT activity compared to both the negative control and BaCl_2_ groups. These biochemical changes may indicate hepatic dysfunction that is not necessarily reflected in macroscopic organ mass.

The brain’s susceptibility to BaCl_2_-induced redox disturbances is well documented [25]. In our study, while no significant lipid peroxidation was observed, the marked activation of antioxidant enzymes (SOD, CAT, GPx) and elevated GSH levels suggest an efficient adaptive response aimed at counteracting potential oxidative insults. The marked induction of SOD in the BaCl_2_ group suggests an upregulation of superoxide detoxification pathways in response to Ba^2+^-induced ROS production. Superoxide anion (O_2_•^−^), a primary ROS generated by impaired mitochondrial electron transport and activation of NADPH oxidases, is efficiently dismutated by SOD into hydrogen peroxide (H_2_O_2_), which requires further detoxification. The concomitant elevation of CAT and GPx indicates an attempt to counteract the increased H_2_O_2_ burden and prevent its conversion into highly reactive hydroxyl radicals via the Fenton reaction, a process particularly detrimental in neural tissues [9].

Interestingly, co-administration of SCC and ASC, either individually or in combination with BaCl_2_, significantly attenuated the induction of SOD, GPx, and GSH compared to BaCl_2_ alone, while CAT activity remained unchanged. These findings suggest that both SCC and ASC effectively reduced the oxidative burden in the brain, likely by directly scavenging ROS and/or chelating redox-active metal ions, thereby lowering the cellular demand for antioxidant enzyme activity. In fact, the potential neuroprotective effect of SCC under oxidative stress conditions has been previously documented. In a model of oxidative stress-induced rats, SCC administration led to a significant reduction in lipid peroxidation in brain tissue, suggesting an effective in situ antioxidant action [25]. However, this protective effect was not associated with modulation of enzymatic antioxidant defenses (SOD, CAT, GPx), but rather with a direct radical scavenging capacity within the cerebral tissue, supporting the notion that SCC can contribute to maintaining cerebral redox homeostasis under prooxidant challenges, through mechanisms complementary to endogenous antioxidant systems. Similarly, ASC exerts multifaceted neuroprotective effects, not only as a direct antioxidant but also as a modulator of redox-sensitive signaling pathways and a cofactor for iron metabolism [26]. It is also plausible that the observed decrease in SOD and GPx activities reflects enzymatic inhibition or exhaustion under sustained oxidative stress, rather than antioxidant efficacy alone [27]. This possibility suggests a threshold beyond which endogenous defenses become overwhelmed or impaired.

The observed decrease in GSH levels in all treated groups, both alone and with BaCl_2_, aligns with the notion that when exogenous antioxidant support reduces ROS generation, endogenous antioxidant systems, including glutathione synthesis and recycling, are downregulated accordingly. This adaptive modulation likely reflects a restoration of redox homeostasis under reduced oxidative stress. Notably, MDA levels remained unchanged between control groups but were significantly decreased in all SCC, ASC, and combination groups under BaCl_2_ exposure, with the most pronounced reduction observed in the combined treatment. This finding underscores the strong protective effect of these molecules against lipid peroxidation in the brain. Given the susceptibility of neuronal membranes to oxidative damage due to a high Polyunsaturated Fatty Acid (PUFA) content, the ability to suppress MDA formation represents a critical aspect of neuroprotection. The enhanced efficacy of the SCC+ASC treatment likely reflects complementary mechanisms of action, where SCC provides metal chelation and membrane stabilization, while ASC contributes potent aqueous-phase ROS scavenging and redox cycling support.

Given the central role of oxidative stress in mediating Ba^2+^-induced hepatic injury, and considering the well-established oxidative susceptibility of the brain [28,29], it is also pertinent to examine whether similar redox alterations and protective mechanisms might be observed at the hepatic level. The liver and brain are metabolically active and highly interconnected organs, and systemic oxidative stress resulting from hepatic dysfunction may further exacerbate neural oxidative damage [30]. Conversely, antioxidant interventions effective in the liver may hold promise for mitigating brain injury under Ba^2+^ exposure. In this context, we next sought to explore the oxidative stress and histopathological profiles of the liver across the same experimental groups.

In the liver, we observed a significant elevation of MDA levels in the BaCl_2_-exposed group, reflecting enhanced lipid peroxidation processes which occurred in the absence of significant modulation of hepatic SOD, CAT, or GPx activities. This lack of enzymatic antioxidant response may reflect an impairment of the Nuclear factor erythroid 2–related factor 2 (Nrf2)-mediated antioxidant pathway [28]. Furthermore, the significant increase in hepatic GSH levels observed in the BaCl_2_ group likely represents an early compensatory response aimed at counteracting excessive ROS accumulation. In fact, the observed ROS accumulation in the liver may stem from NOX upregulation, particularly NOX2 (pro-inflammatory) or NOX4 (fibrogenic) [8]. Measuring NOX subunit expression (e.g., p47phox for NOX2) or activity could identify the primary ROS source. Pharmacological NOX inhibition (e.g., apocynin) could further dissect their contribution to Ba^2+^ toxicity. Nevertheless, histopathological analysis further corroborated these biochemical findings, revealing severe hepatic lesions in the BaCl_2_ group, including marked inflammation and areas of necrosis. These alterations are consistent with the literature, where BaCl_2_-induced hepatotoxicity has been linked to oxidative stress-driven tissue injury [1]. The pronounced increase in lipid peroxidation, coupled with the absence of a coordinated enzymatic antioxidant response, suggests that alternative prooxidant pathways may contribute to ROS production in this context. In particular, activation of hepatic NADPH oxidases (NOX isoforms), which are well expressed in hepatocytes and non-parenchymal liver cells, may play a central role. NOX1 and NOX2 primarily generate superoxide anions (O_2_•^−^), which are rapidly dismutated to hydrogen peroxide (H_2_O_2_), while NOX4 directly produces H_2_O_2_ as its main product [29]. Excessive accumulation of these ROS can overwhelm antioxidant defenses, promote lipid peroxidation, and impair Nrf2-mediated transcription of antioxidant enzymes, a mechanism well characterized for several divalent metal ions and suggested for barium in emerging studies [1]. Under such conditions, it is also plausible that the cellular response shifted away from an adaptive antioxidant strategy toward pathways of cell death, as supported by our histopathological findings, which revealed extensive inflammation, necrosis, and structural damage. This phenomenon is consistent with the concept of a “point of no return” in oxidative stress, where excessive ROS accumulation induced irreversible damage that leads to cell death through various pathways, such as apoptotic, necrotic, or ferroptosis, rather than engaging protective enzymatic responses [10,30].

Interestingly, SOD activity was significantly decreased in all groups co-treated with antioxidant compounds and BaCl_2_, as well as in the SCC-alone group. This finding may reflect the capacity of SCC and ASC to directly neutralize ROS, thereby attenuating the redox signals necessary for Nrf2 activation and subsequent SOD expression. In addition, SCC possesses metal-chelating properties capable of interacting with biologically relevant metal ions (e.g., Fe^3+^, Cu^2+^, Zn^2+^). While other metal chelators are known to impair Cu/Zn-SOD activity [31], direct evidence of SCC producing such an effect remains limited and largely inferred from analogous mechanisms. In contrast, both GPx and CAT activities remained unchanged. This enzymatic stability suggests that the redox shifts induced by BaCl_2_ and the antioxidant treatments were insufficient to trigger further modulation of these enzymatic defenses under the present experimental conditions.

Regarding lipid peroxidation, the combined administration of SCC and ASC with BaCl_2_ led to a significant reduction in hepatic MDA levels compared to the BaCl_2_ group alone, demonstrating that both compounds effectively mitigated lipid oxidative damage.

An additional point of interest in our study concerns the modulation of hepatic GSH levels. Both SCC, ASC, and their combination, when administered alone, resulted in significant GSH depletion compared to the negative control group. Under BaCl_2_ exposure, the treated groups also significantly decreased GSH levels relative to the BaCl_2_ group. These observations likely reflect enhanced GSH consumption driven by the redox cycling of ASC and by SCC-induced modulation of GSH metabolism.

Histopathological findings provided complementary insights into these biochemical outcomes. The SCC-alone group exhibited normal liver architecture, indicating good hepatic tolerance. In contrast, the ASC-alone group displayed centrilobular vacuolization and signs of hepatocellular distress, consistent with localized prooxidant effects of high-dose ASC reported in the literature [18]. In the BaCl_2_ + SCC group, mild protective effects were evident, with light vacuolization but without severe structural damage. Conversely, the BaCl_2_ + ASC group exhibited pronounced vacuolization, necrosis, and strong inflammatory infiltration, suggesting that ASC alone was insufficient to protect against BaCl_2_-induced injury and may have exacerbated certain pathological features. The treatment of SCC + ASC under basal conditions preserved normal liver histology, confirming the good tolerance of this treatment in the absence of exogenous oxidative stress. However, in the BaCl_2_ + SCC + ASC group, a severe inflammatory response resembling autoimmune hepatitis was observed, highlighting a complex and potentially adverse immunomodulatory interaction between these compounds in the context of Ba^2+^-induced oxidative stress.

Altogether, these findings highlight the complex interplay between prooxidant and antioxidant mechanisms in Ba^2+^-induced toxicity, and underscore the potential, yet context-dependent, benefits and risks of SCC and ASC as protective agents. Further studies are warranted to elucidate the immunomodulatory interactions of SCC and ASC—both individually and in combination, and to optimize their protective effects against heavy metal-induced oxidative stress, including potential impacts on other organs.

## 4. Materials and Methods

### 4.1. Chemicals and Reagents

All chemicals were purchased from Sigma Aldrich (St. Louis, MO, USA) unless otherwise stated.

### 4.2. Animals

Male and female Swiss albino mice were used in this study, and all procedures were carried out following the instructions of the Institutional Animal Ethics Committee of Moroccan Association For Research and Ethics (Animal Ethics code: 11-REC-23). Mice were housed in an air-conditioned animal room under a 12 h light/dark cycle at 24 ± 5 °C and 55 ± 10% Relative Humidity (RH) and provided a laboratory diet and water ad libitum.

### 4.3. Toxicological Study Design

A total of 60 healthy adult Swiss mice (both sexes, 12 weeks old, weighing 26–30 g) were used for the subacute toxicity of SCC, ASC, and their combination. After a 7-day acclimatization period, the mice were randomly assigned to one of the following treatment groups for a subacute toxicity study: SCC (40 mg/kg), ASC (160 mg/kg), or a combination of both. Compounds were administered once daily for 14 consecutive days. Male and female mice were housed separately, and only non-pregnant, nulliparous females were included. All experimental procedures were conducted in compliance with ethical standards and were approved by the Institutional Animal Ethics Committee.

#### 4.3.1. Behavioral and Neurotoxicity Assessments

To evaluate potential neurobehavioral toxicity, mice were subjected to a battery of validated behavioral tests. The Irwin test (1968) was employed to screen for changes in general physiology and behavior, including motor activity, anxiety-like responses, memory, and sensorimotor coordination. Observations were carried out systematically according to standardized protocols at the end of the treatment period. Anxiety and exploratory behavior were further assessed using the Open Field Test, conducted in a 100 × 100 × 40 cm wooden arena divided into 25 squares and illuminated by a 100 W lamp. After a 1-minute acclimation, each mouse was video-tracked for 10 minutes using Anymaze software (Stoelting Company, Wood Dale, IL, USA. Key endpoints included time spent in the central zone (an index of anxiety) and the number of grid crossings (locomotor activity). The arena was thoroughly sanitized with 70% ethanol between sessions.

#### 4.3.2. Cognitive and Motor Function Evaluation

Cognitive performance was assessed using the novel object recognition (NOR) test conducted in the same arena. The protocol included three sequential phases: habituation, acquisition (5-minute exploration of two identical objects), and retention, in which one object was replaced by a novel one after a 1-hour delay. The discrimination index (DI) was calculated as follows:

(1) $$DI=\;\frac{TimeNovel-TimeFamiliar}{TimeNovel+TimeFamiliar}$$

All objects and surfaces were cleaned with 70% ethanol after each session to eliminate olfactory cues. In addition, motor coordination and balance were evaluated using the rotarod test (7 cm rod, 30 rpm). Each animal underwent five trials (maximum 3 min/trial), with a 2 min inter-trial interval. The primary outcome was latency to fall, recorded for each trial. The apparatus was sanitized between uses to prevent cross-contamination or scent marking.

### 4.4. Animals and Experimental Design

Male *Swiss albino* mice (8–10 weeks old, 25–30 g body weight) were used in this study. Regular body weight monitoring was carried out during the entire housing duration. Following a 10-day acclimatization period, the mice were randomly separated to eight groups (4-6 mice per group). Notably, the control and treated (─BaCl_2_) groups consisted of 4 mice, while all treated groups included 6 mice each (Table 2). The doses were determined based on prior in vivo antioxidant studies using similar biochemical and histological endpoints [32,33]. Although no formal power analysis was performed, the group sizes ensured observable biological responses, while adhering to ethical use of animals.

**Figure 10:**
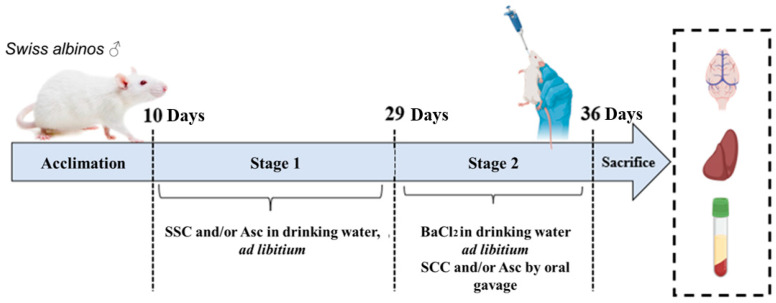
In vivo experimental design. Male *Swiss albino* mice were acclimatized for 10 days prior to treatment. During stage 1 (days 10 to 29), mice received SCC and/or ASC in drinking water ad libitum. During stage 2 (days 29 to 36), BaCl_2_ was administered in the drinking water to induce oxidative stress and inflammation, while SCC and/or ASC treatments were maintained by oral gavage. On day 37, the animals were sacrificed, and brain, liver, and blood samples were collected for biochemical and histopathological analyses.

The experimental design followed a two-stage process (Figure 10):

**Stage 1:** For 19 days, control and BaCl_2_ groups were given plain water, SCC and SCC + BaCl_2_ groups were given SCC (40 mg/L) in drinking water ad libitum, ASC and ASC + BaCl_2_ groups were given L-ascorbic acid (3.3 g/L) in drinking water ad libitum, and SCC + ASC and SCC + ASC + BaCl_2_ groups were given a mixture of SCC and L-ascorbic acid in drinking water ad libitum.

**Stage 2:** One week before euthanasia, BaCl_2_ groups were given 150 mg/L of BaCl_2_ in drinking water ad libitum, which corresponds to approximately 1/12 to 1/20 of the reported oral LD_50_ (300–500 mg/kg body weight in mice [34]. SCC/L-ascorbic acid supplementation was switched to gavage at 40 mg/kg/160 mg/kg, respectively. The switch was performed to avoid variability in antioxidant intake caused by potential changes in water consumption during BaCl_2_ exposure, and SCC and ASC administration was shifted from drinking water to oral gavage during the final week to ensure precise dosing.

On day 37, mice were humanely sacrificed, and blood was collected in separate tubes for serum biochemical analysis. Non-heparinized blood was centrifuged at 1100× *g* for 10 min, and the supernatant was collected and stored at 4 °C. Liver and brain were immediately collected and weighed. The liver was divided into two portions: one was rinsed with 0.9% NaCl and fixed in 10% buffered formalin for histopathological analysis, while the other was stored at −80 °C for enzymatic assays. The brain was directly preserved at −80 °C for biochemical analysis only. Humane endpoints were established based on clinical signs such as severe weight loss, lethargy, or respiratory distress. Animals were monitored daily throughout the experiment. No animals met these criteria during the study.

### 4.5. Serum Biochemical and Ionic Parameters

Blood samples were collected in a red top tube and centrifuged to obtain serum, which was then analyzed using an automated biochemistry analyzer (COBAS INTEGRA 400, Roche Diagnostics, Rotkreuz, Switzerland). This analyzer is widely used in clinical laboratories to measure a broad range of biochemical and ionic parameters in serum samples, employing both photometric methods (for enzymes, proteins, and metabolites) and ion-selective electrode (ISE) technology (for electrolytes) [35]. The following parameters were quantified: triglycerides (g/L), urea (mg/L), creatinine (mg/L), uric acid (mg/L), total protein (g/L), cholesterol (g/L), aspartate aminotransferase (ASAT, IU/L), alanine aminotransferase (ALAT, IU/L), total bilirubin (mg/L), albumin (g/L), sodium (Na^+^, mmol/L), potassium (K^+^, mmol/L), calcium (Ca^2+^, mg/L), and chloride (Cl^−^, mmol/L). All measurements were performed according to the manufacturer’s protocols, with internal quality controls ensuring assay precision and accuracy.

### 4.6. Homogenate Preparation 

Brain and liver homogenates (20% w/v) were prepared in 50 mM phosphate buffered saline (PBS) (KH_2_PO_4_, K_2_HPO_4_, pH 7.4) Using a Potter-Elyehiem homogenizator (Millville, NJ, USA). The resulting homogenates were then sonicated for 10 s in three cycles using an OMNI SONIC RUPTOR 4000 to ensure full cell disruption, then centrifuged at 3000× *g* for 10 min at 4 °C, and the resultant supernatant was removed and stored at −20 °C. Protein concentration was determined following the Bicinchoninic Acid Assay [36], employing bovine serum albumin (BSA) as a protein standard.

### 4.7. Protein Content

Protein content was measured using bovine serum albumin as a standard, according to the method described by [37]. The absorbance was read spectrophotometrically at 750 nm (infinite M200 PRO, TECAN, Zürich, Switzerland).

### 4.8. Superoxide Dismutase

As described by Beyer et al [38], the method is a colorimetric test commonly used to assess the enzymatic activity of superoxide dismutase (SOD). It is based on the enzyme’s capacity to convert nitroblue tetrazolium (NBT) to formazan. It measures total SOD activity, including all the isoforms of the enzyme contained in the sample. Using a 96-well plate, a reaction mixture was prepared including 14 µL homogenate, 86 µL PBS (116 mM, pH 7.4), 20 µL Triton X-100 (0.025%), 20 µL EDTA (0.1 mM, pH 8.0), 20 µL L-methionine (12 mM), 20 µL NBT (75 mM), and 20 µL riboflavin (2 µM). After mixing, a 15 W light source was shone on the plate for 10 min to initiate the photochemical reactions. Then, absorbance was measured at 560 nm using a TECAN Infinite M 200 PRO spectrophotometer, to assess SOD activity.

### 4.9. Catalase

Catalase (CAT) activity was measured by photometric assessment of H_2_O_2_ degradation at 240 nm using homogenate as the enzyme source [39]. This reaction was performed in a 96-well UV-specific microplate. Following the reaction, each well included a reaction mixture of 10 µL of H_2_O_2_ (400 mM), 10 µL of PBS (1 M) and 170 µL of distilled water, to which 10 µL of the sample homogenate was added. CAT activity was represented by the quantity of enzyme needed to degrade 1 µmol of H_2_O_2_ per minute.

### 4.10. Lipid Peroxidation

Malondialdehyde (MDA), a sub-product of lipid peroxidation, ranks as one of the most frequently cited biomarkers for monitoring free radical-induced damages. The method is essentially based on the creation of a pigmented complex involving one molecule of MDA and two molecules of thiobarbituric acid (TBA). A total of 25 µL of homogenate was added to 25 µL of trichloroacetic acid (TCA, 5%) as well as 50 µL of TBA (0.67%). The resulting mixture was heated to 100 °C for 15 min. After freezing, 200 µL of n-butanol was added. The organic layer was separated by centrifugation at 3000× *g* for 15 min. The absorbance of the supernatant was determined at 532 nm using a spectrophotometer [37,40].

### 4.11. Glutathione

Reduced glutathione (GSH) plays a vital role as an antioxidant at the cell level, in both redox homeostasis and the protection of cells versus oxidative damage. The Ellman approach to GSH measurement is designed on the basis of the interaction of GSH with 5,5’-dithiobis (2-nitrobenzoic acid) (DTNB) to generate a chromogenic product that can be measured spectrophotometrically [41,42]. Under this assay, DTNB specifically acts on the thiol group (-SH) of GSH to generate glutathione disulfide (GSSG) and liberate the anion 5-thio-2-nitrobenzoate (TNB-). Reduced glutathione (GSH) levels were determined by measuring the absorbance of 5-thio-2-nitrobenzoic acid (TNB^−^), the yellow-colored product formed from the reaction of GSH with 5,5′-dithiobis (2-nitrobenzoic acid) (DTNB), at 412 nm. To prepare the samples, 60 µL of tissue homogenate was mixed with 30 µL of 5% trichloroacetic acid (TCA) to precipitate proteins. The mixture was centrifuged at 12,000× *g* for 10 min at 4 °C, and 40 µL of the resulting supernatant was transferred to a new well and combined with 140 µL of PBS (50 mM, pH 8.0) and 20 µL of DTNB solution (6 mM). After incubation at room temperature for 5 min, absorbance was measured at 412 nm using a spectrophotometer. A standard curve was generated using known concentrations of GSH reacted with DTNB under the same conditions to calculate the GSH concentration in the samples.

### 4.12. Glutathione Peroxidase

Total glutathione peroxidase (GPx) activity was assessed based on the enzyme’s ability to oxidize GSH [43], which subsequently reacts with 5,5′-dithiobis (2-nitrobenzoic acid) (DTNB) to produce a yellow-colored compound, 5-thio-2-nitrobenzoic acid (TNB), measurable at 412 nm. The reaction mixture for the GSH-containing sample consisted of 15 µL PBS (0.1 M, pH 7.0), 10 µL GSH (30 mM), 5 µL sodium azide (10 mM), and 15 µL tissue homogenate, incubated at 37 °C for 15 min. A control sample without GSH (replaced by PBS) was used to subtract the absorbance of endogenous GSH. A standard without homogenate was prepared to assess the total GSH content in the absence of enzymatic activity and to account for any background absorbance from the reagents. An additional blank lacking both GSH and homogenate was included to eliminate interference from other reactive components. To quantify the concentration of GSH, a standard curve was established using known concentrations of GSH reacted with DTNB under the same conditions. After incubation, reactions were stopped by adding 5% trichloroacetic acid (TCA), followed by centrifugation at 1500× *g* for 5 min. Finally, 40 µL PBS (50 mM) and 140 µL DTNB solution (0.04%) were added to the supernatant, and absorbance was measured spectrophotometrically at 412 nm.

### 4.13. Histopathological Analysis

According to RITA guidelines [44,45], liver tissue was fixed in 10% buffered formaldehyde, embedded in paraffin, and sectioned and stained with hematoxylin and eosin (H&E) for histological analysis. Brain tissue was not subjected to histopathological examination due to the technical challenges of maintaining neural cytoarchitecture integrity with our fixation protocol, which was optimized for hepatic tissue preservation. 

The assessment was performed by a board-certified pathologist (HA) in blinded conditions to the experimental groups. Tissues stained with hematoxylin and eosin (H&E) were examined for hepatocellular alterations including inflammatory infiltration, sinusoidal congestion, necrosis, and structural disorganization. Observations were described qualitatively based on predefined criteria. Each tissue slide was scanned using the VENTANA DP 200 slide scanner (Roche Diagnostics, Tucson, AZ, USA), then subjected to detailed examination and imaging using the online PATHOMATION software (Pathomation BV, Amsterdam, Netherlands) platform.

### 4.14. Statistical Analysis

Statistical analysis was performed using GraphPad Prism 9.4.0 software (GraphPad Software Inc., San Diego, CA, USA). The two-way ANOVA test was used to analyze multiple groups, followed by Tukey’s multiple comparisons test. Differences were considered statistically significant at *p* < 0.05.

## 5. Conclusions and Perspectives

This study demonstrates that BaCl_2_ exposure triggers distinct oxidative responses in the liver and brain—inducing hepatic injury with lipid peroxidation and GSH elevation but activating adaptive antioxidant defenses in the brain. SCC showed protective effects in both organs, while ASC exhibited prooxidant tendencies in the liver. Combining SCC and ASC reduced oxidative damage but exacerbated hepatic inflammation, highlighting organ-specific risks. Nevertheless, while this study advances our understanding of organ-specific antioxidant protection against BaCl_2_ toxicity, several limitations warrant consideration. The relatively small sample size and brief exposure period may affect statistical power and obscure longer-term effects, emphasizing the need for extended investigations with larger cohorts. The absence of brain histopathological analysis represents a missed opportunity to correlate biochemical markers with structural changes, while more comprehensive neurobehavioral assessments could have provided deeper functional insights. Looking ahead, future research should explore the intriguing prooxidant behavior of ascorbic acid in Ba^2+^-rich environments and rigorously test sodium copper chlorophyllin’s potential metal-chelating properties through direct binding assays. Mechanistic studies could profitably examine Nrf2 pathway activation patterns across different tissues, along with detailed characterization of inflammatory responses. Dose–response relationships also merit careful evaluation to establish optimal therapeutic windows, potentially revealing how to maximize protective effects while minimizing adverse interactions. These investigations would not only address current knowledge gaps but also strengthen the translational relevance of these findings for clinical and environmental applications.

## Figures and Tables

**Table 1:** Serum biochemical and ionic parameters of each experimental group: control, SCC, ASC, and SCC + ASC, under both BaCl_2_-untreated (*(−) BaCl*_2_) and BaCl_2_-treated (*(+) BaCl*_2_) conditions. Data are expressed as mean ± SD. *n* = 4 for (−) BaCl_2_ groups and *n* = 6 for (+) BaCl_2_ groups. Data are expressed as mean ± SD. Statistical significance: ^#^ p < 0.05 vs. BaCl_2_ group, * p < 0.05 vs. control group.

	Control	BaCl_2_	SCC	SCC + BaCl_2_	ASC	ASC + BaCl_2_	SCC + ASC	SCC + ASC + BaCl_2_
**Triglyceride (g/L)**	0.54 ± 0.06	0.54 ± 0.05	0.60 ± 0.07	0.57 ± 0.07	0.6 ± 0.04	0.6± 0.14 ^#^	0.55± 0.03	0.66 ± 0.06
**Urea (mg/L)**	0.15 ± 0.03	0.19 ± 0.03	0.16 ± 0.02	0.2 ± 0.03	0.19 ± 0.02	0.2 ± 0.02	0.18 ± 0.01	0.19 ± 0.02
**Creatinine (mg/L)**	3.68 ± 0.84	3.23 ± 0.68	2.49 ± 1.04	2.83 ± 0.63	3.54 ± 0.96	3.47 ± 0.51	2.94 ± 0.43	3.75 ± 0.72
**Uric acid (mg/L)**	23.43 ± 2.51	27.83 ± 5.23	30.75 ± 1.82	25.22 ± 2.64	27.67 ± 2.48	27.03 ± 2.82	25.86 ± 4.39	30.36 ± 2.1
**Total protein**	64.89 ± 0.92	57.78 ± 3.03	64.84 ± 2.85	61.6 ± 5.91	62.38 ± 3.67	63.3 ± 3.43	61.67 ± 2.27	56.03 ± 5.17
**Cholesterol (g/L)**	0.84 ± 0.1	0.83 ± 0.04	0.84 ± 0.05	0.87 ± 0.04	0.76 ± 0.08	0.83 ± 0.07	0.85 ± 0.03	0.91 ± 0.09
**ASAT (IU/L)**	163.44 ± 3.91	173.41 ± 4.75	189.52 ± 15.83	175.33 ± 13.31	195.82 ± 48.98	250.93 ± 77.49 ^*,#^	178.58 ± 4.66	175.27 ± 10.6
**ALAT (IU/L)**	32.86 ± 11.85	27.92 ± 5.47	35.91 ± 10.33	32.8 ± 10.72	35.49 ± 7.85	39.71 ± 8.83	32.62 ± 3.42	30.24 ± 1.97
**Total bilirubin (mg/L)**	0.49 ± 0.18	0.38 ± 0.07	0.47 ± 0.18	0.37 ± 0.09	0.49 ± 0.1	0.4 ± 0.13	0.4 ± 0.04	0.33 ± 0.06
**Albumin (g/L)**	48.44 ± 3.71	63.43 ± 5.04	64 ± 2.39	61.16 ± 10.76	58.21 ± 10.44	62.39 ± 8.95	58.01 ± 9.34	69.83 ± 0.91
**Na^+^ (mmol/L)**	134.42 ± 1.92	134.58 ± 1.16	136.39 ± 1.98	134.31 ± 1.45	134.47 ± 2.56	133.23 ± 1.67	134.49 ± 0.85	133.26 ± 2.28
**K^+^ (mmol/L)**	5.4 ± 0.14	5.2 ± 0.32	5.23 ± 0.13	5.15 ± 0.49	5.10 ± 0.57	4.88 ± 0.3	5.25 ± 0.19	4.92 ± 0.47
**Ca^2+^ (mg/L)**	103.34 ± 3.01	98.62 ± 3.14	100.99 ± 2.31	102.73 ± 3.23	100.70 ± 1.15	100.51 ± 4.44	101.03 ± 2.15	99.19 ± 3.81
**Cl^─^ (mmol/L)**	94.44 ± 2.85	95.22 ± 2.04	96.6 ± 2.24	98.02 ± 2.56	97.07 ± 3.88	97.37 ± 2.05	96.81 ± 1.43	98.79 ± 1.73

**Table 2:** Experimental design and treatments administration.

Group	Diet	SCC and ASC Supplementation	BaCl_2_ Supplementation
Control	Standard diet	Plain water	No
BaCl_2_			150 mg/L
SCC		In drinking water (40 mg/L) By gavage (40 mg/Kg)	No
SCC + BaCl_2_			150 mg/L
Asc		In drinking water (3.3 g/L) By gavage (160 mg/Kg)	No
Asc + BaCl_2_			150 mg/L
SCC + Asc		**SCC:** In drinking water (40 mg/L) By gavage (40 mg/Kg) **ASC:** In drinking water (3.3 g/L) By gavage (160 mg/Kg)	No
SCC + Asc + BaCl_2_			150 mg/L

## Data Availability

Data are contained within the article.

## Abbreviations

The following abbreviations are used in this manuscript:

BaCl_2_	Barium chloride
SCC	Sodium copper chlorophyllin
ASC	Ascorbic acid (Vitamin C)
ROS	Reactive oxygen species
MDA	Malondialdehyde
GSH	Glutathione
SOD	Superoxide dismutase
CAT	Catalase
GPx	Glutathione peroxidase
ETC	Electron transport chain
Nrf2	Nuclear factor erythroid 2-related factor 2
NOX	NADPH oxidase
H_2_O_2_	Hydrogen peroxide
O_2_•^−^	Superoxide anion
DTNB	5,5′-dithiobis (2-nitrobenzoic acid)
TNB^−^	5-thio-2-nitrobenzoate
TCA	Trichloroacetic acid
BSA	Bovine serum albumin
H&E	Hematoxylin and eosin
RITA	Registry of Industrial Toxicology Animal-data
NACAD	National Advisory Committee for Acute Exposure Guidelines
ANOVA	Analysis of variance
LD_50_	Lethal dose, 50%
